# Materials discovery of ion-selective membranes using artificial intelligence

**DOI:** 10.1038/s42004-022-00744-x

**Published:** 2022-10-20

**Authors:** Reza Maleki, Seyed Mohammadreza Shams, Yasin Mehdizadeh Chellehbari, Sima Rezvantalab, Ahmad Miri Jahromi, Mohsen Asadnia, Rouzbeh Abbassi, Tejraj Aminabhavi, Amir Razmjou

**Affiliations:** 1 Department of Chemical Engineering, Shiraz University, Shiraz, Iran; 2 Department of Petroleum Engineering, Amirkabir University of Technology, Tehran, Iran; 3 Department of Chemical Engineering, Sharif University of Technology, Tehran, Iran; 4 Renewable Energies Department, Faculty of Chemical Engineering, Urmia University of Technology, 57166-419 Urmia, Iran; 5 School of Engineering, Macquarie University, Sydney, NSW 2109 Australia; 6 School of Engineering, Faculty of Science and Engineering, Macquarie University, Sydney, NSW Australia; 7 School of Advanced Sciences, KLE Technological University, Hubballi, Karnataka 580 031 India; 8 Mineral Recovery Research Center (MRRC), School of Engineering, Edith Cowan University, Joondalup, Perth, WA 6027 Australia; 9 UNESCO Centre for Membrane Science and Technology, School of Chemical Engineering, University of New South Wales, Sydney, NSW 2052 Australia

**Keywords:** Computational chemistry, Materials chemistry, Porous materials

## Abstract

Significant attempts have been made to improve the production of ion-selective membranes (ISMs) with higher efficiency and lower prices, while the traditional methods have drawbacks of limitations, high cost of experiments, and time-consuming computations. One of the best approaches to remove the experimental limitations is artificial intelligence (AI). This review discusses the role of AI in materials discovery and ISMs engineering. The AI can minimize the need for experimental tests by data analysis to accelerate computational methods based on models using the results of ISMs simulations. The coupling with computational chemistry makes it possible for the AI to consider atomic features in the output models since AI acts as a bridge between the experimental data and computational chemistry to develop models that can use experimental data and atomic properties. This hybrid method can be used in materials discovery of the membranes for ion extraction to investigate capabilities, challenges, and future perspectives of the AI-based materials discovery, which can pave the path for ISMs engineering.

## Abstract (web summary)

Ion separation membranes are of importance for a range of applications, including water treatment, raw material recovery, gas separation, and fuel cells, but traditional research and development methods can be expensive and time-consuming. Here, the authors review the capabilities and limitations of artificial intelligence in the design of high performing ion-selective membranes.

## Introduction

The traditional research and development (R&D) methods can hardly fulfill the ever-growing demand for innovative materials and energy resources. The outpaced R&D methods are delayed by several factors, such as arduous and expensive experiments and time-consuming computer simulations^1^. These empirical and computational approaches can contribute to each step of the multi-stage conventional methods (e.g., discovery, development, property optimization, etc.)^2,3^. Considering probable iteration between stages, novel chemicals and resources will be held well behind the soaring demand^4^. During recent decades, advances in various sciences have led to an increased focus on energy-efficient materials and methods, especially membrane separation technology. Membrane technology, given its simplicity, scalability, and small footprint, has been a focus for many applications, including water and wastewater treatment, gas separation, filtration, pharmaceuticals, batteries, and fuel cells^5^. Ion separation becomes essential for the mentioned applications. Ion-selective membranes (ISMs) are utilized into various processes, from biological membranes to industrial separations. ISMs enable the recovery of raw materials from natural resources and/or wastewater sources^6–8^.

While handling a large amount of data with multiple steps, neither the traditional experimental nor the computational approaches are feasible. To accelerate the data management, artificial intelligence(AI), with its inherent capabilities in the handling of a large amount of data, seems applicable in various fields such as drug discovery^9,10^, disease diagnosis^11–13^, advanced energy materials^14^, catalysts^15,16^, gas and oil industry^17,18^. Associated limitations of the conventional methods (like time-consuming experimental and computational works, high experimental work costs, unsafe work conditions with toxic materials, and high pressure and temperature conditions) accentuate rapid and accurate methods. AI and its subgroups are proven effective tools for finding quick and efficient solutions under various situations. For instance, KiJeon Nam et al.^19^ used Deep Learning to evaluate the optimal conditions for an effluent treatment unit, including membrane bioreactor (MBR), and by using the optimal conditions, they could save up to 4% on the energy consumption of this unit. Besides that, using AI for material discovery can be very useful. New material discovery dating back to the human history that started in the Stone Age and continuing till date^20^. The AI-related techniques in material discovery are investigated by Yang et al.^21^ together with a brief history of AI development. Accelerating the discovery of new materials and maturing and implementing these technologies into deployment will require a radical departure from the traditional forms of discovery. It also requires a broad effort that brings a different variety of individuals working across their specialties. Hence, material discovery and development cross-cutting the entire separation technology portfolio from membranes and zero-carbon emissions to valuable metal separation, delivery, and other end-uses.

**Fig. 1:**
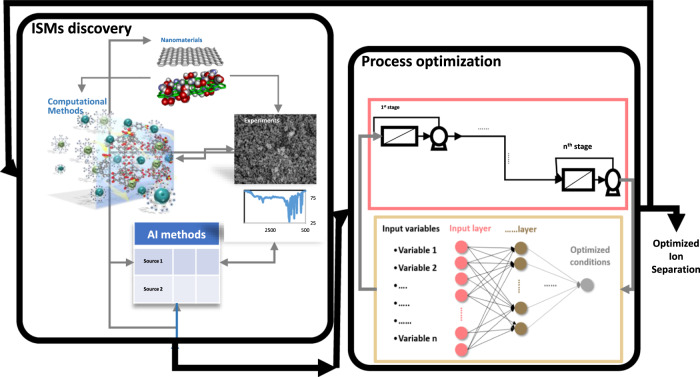
Schematic representation of the application of AI methods for the discovery and design of new ISMs. AI can optimize current ISMs or suggest new ISMs based on computational (or experimental) data. Optimization and discovery of new materials in this method are based on a model that AI builds from related computational (or experimental) datasets. Based on this model, AI can determine the optimal conditions for ion separation (including ISMs type).

One of the most important areas that can use the capabilities of AI for material discovery is membranes that are used to extract valuable metal ions^22,23^. The growing demand for purification methods has developed the intense applications of AI and machine learning (ML) in ISMs’ progress. A model trained by AI and ML provides an efficient avenue to reduce computational power consumption. AI and its subsets have been extensively used in several membrane separation technologies^24–26^. As shown in Fig. 1, the AI methods can be utilized in every step of the valuable metal ions separation process, the same as Li separation, including discovering the new materials^27^, optimizing process parameters^19,28,29^, and finally improving the existence of metal ions extraction methods^30–33^. Consequently, in the presence of rich databases, intelligent methods can take a further step and interfere in discovering the new ISMs. In this sense, AI and ML can help researchers improve the selectivity of ISMs without sacrificing permeability. It can be the dawn of a new era in membrane technologies and purification processes.

In the previous works, the performance of some ISMs has been investigated using AI tools. However, no comprehensive study of the application of AI in ISMs material discovery has been reported. In fact, only some limited works have been performed on some case studies. Given the growing use of AI in this field, a review work that can comprehensively study the application of AI in ISMs material discovery can be of great help to further research. Having such review work, subsequent studies using AI tools can be performed more accurately, and better perform ISMs material discovery. The current review aims to critically discuss the immature development of AI and ML techniques in membrane technologies, especially in designing and discovering new ISMs with higher selectivity and permeability towards the desired ions. In this regard, we review the limitations and capabilities of AI and ML that could be used to design more efficient ISMs. Finally, using atomic properties obtained from MD and DFT calculations, one can pave the path for developing AI and ML approaches to discover more efficient ISMs.

## Data preprocessing and AI feature engineering

One of the essential steps of material discovery is data gathering. Generally, the volume and reliability of data conduct the ML results to the practical and direct path. Therefore, data preprocessing and feature engineering are needed. By implementing these two steps, machines can better understand the material specification relationships and optimize the process parameters and material predicting models to improve the existence of valuable metal ions extraction methods^1,20,34^. Data collection and data cleaning are the two main steps of data preprocessing, and there are many types of data collection, but researchers need to collect representative data. It would be most reasonable to choose appropriate data for specific problems. Accordingly, to reach an accurate and efficient predicting model to improve the efficiency of data analysis, data cleaning is necessary to be free from irrelevances and incorrect information^1^.

In order to build a complete ML dataset, one can select and mine the appropriate data relay on high-throughput theoretical simulations from the existing database. Some open-source databases generally provide guidelines, standards, or recipes to facilitate any new product’s preparation. The MGI project^1,34^ in 2011 proposed a comprehensive environment for researchers to deepen their investigation of materials and build a unified database to predict new materials’ properties. The open-source databases such as AFLOWLIB and Materials Project are the global database and power analysis tools for researchers, particularly for inorganic materials^35,36^such as in Harvard Clean Energy Project^37^, ZINC, and GDB^38^ for organic molecules and organic solar cell materials. On the other hand, NanoHUB is an open-source database focusing on nanomaterials and Open Quantum Materials, and Cambridge Structural Databases^36^, which contain a substantial amount of data on the structural properties of materials, are a good choice for sample input data for the ML approach. Also, Atomic Simulation Environment, Python Materials Genomics (pymatgen)^39^, Automated Interactive Infrastructure and Database (AiiDA)^40^ are the open-source automated environments that could provide many simulation tasks.

The first vital issue in material discovery is sufficient knowledge about the features. Different mechanism of the various membranes leads to multiple application areas and features. Therefore, the machine must interpret the input and output data of the ML infrastructure through the modified learning process^20^. Accordingly, feature engineering presents the most precise correlation between the features and labels to approach the best performance. The following parts will introduce the two main steps of data preprocessing and the essential features in the material discovery of ISMs. feature the selection and how they can function in ISMs discovery as artificial intelligent (AI) tools for feature engineering.

### Feature selection in materials discovery of ISMs

The choice of input and output data in ML procedures is crucial. The machine should utilize and benefit from the data integration to reconstruct the datasets and ensure a meaningful correlation between the volume and reliability of data entering and exiting the ML infrastructure^1^. In the predictive modeling process, feature selection is a useful tool for identifying and removing irrelevant data and selecting appropriate variables from those that do not contribute to the accuracy of the models, resulting in better accuracy in the model^1^.

**Fig. 2:**
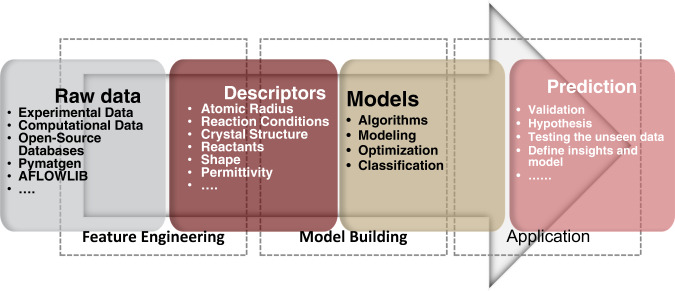
Stages of AI-based ISM discovery. The main descriptors should be selected from the raw datasets. This step is performed using feature engineering. Data screening, determination of input, output and, communicated descriptors are done in this step. After that, AI can train models that can predict the performance of different ISMs. The accuracy and reliability of the model are crucially dependent on feature engineering.

As shown in Fig. 2, descriptors are the appropriate features extracted from the process of feature engineering. Feature engineering helps to develop some meaningful descriptors that are relevant to the output. As a result, the quality of predicting model would be related to the quality of significant input material-specific features. Therefore, creating a set of meaningful descriptors is crucial. Currently, manually creating a set of significant features depending on the properties of experimental studies and using relevant mathematical and physical assessments to implement those features into numerical vectors are the two main ideas for determining descriptors for the experimental candidates’ properties^1,41^. Ghiringhelli et al.^41^ consider four primary standards for a descriptor with the minimum dimensions, characterizing the material the same as the property-relevant elementary process, characterizing and selecting materials based on similar descriptor values, and the simplicity of determining the descriptors^41^.

The purpose here is to highlight and collect the trends in research progress related to the ISMs. Mainly, ion transport mechanisms inside the nanochannels play essential roles in designing ISMs. The main highlight is to emphasize the fundamental concept of ion transport within the membranes and achieve better results are nanochannels size and geometry, material design, and fabrication methods.

#### Descriptors for ISMs

In the process of feature engineering, a large number of data has been accumulated from the experimental and computational investigations that are incomplete, complicated, redundant, and inconsistent. Therefore, data cleaning and data preprocessing need to be undertaken to develop an efficient ML predicting model and reduce the extent of calculation. In order to express different mechanisms, organize independent variables, and find relationships between the hidden data and other meaningful features, high-performance descriptors should be designed. As the membranes are well-known for their size, distance, surface charge, chemistry and morphology of the nanochannels, and driving force, they have been actively utilized for ion transport mechanism, especially for predicting the ions selectivity^7^. In order to achieve practical applications, suitable descriptors must be chosen depending on the different situations such as discussed below.

##### Effect of nanochannel size

During the membrane fabricating process, high permeability, high ion selectivity, controlling the size of the nanopores, and providing a large number of uniformly sized nanopores, should be considered crucial parameters^7,42^. The nanochannel size is considered the most critical parameter in controlling ion selectivity. Increasing the size of the nanochannels decreases ion selectivity. The dehydration process has a crucial impact on ion selectivity when the surface of nanochannels is neutral. The separation of ions thus occurs when the ions lose part of their hydration layer to enter the membranes, which means that the size of the nanochannels must be smaller than the hydration ionic diameter^43–45^. Hence, for the non-charged nanochannels with less than 0.74 nm, alkali metal ions must lose some of the associated water molecules or part of their hydration layer to enter the channel^41^.

The monovalent ion selectivity becomes more critical when there is no charge on the nanochannels walls. Abraham et al.^46^ prepared stabilized graphene oxide membranes with a pore size smaller than the hydration ionic diameter to observe that reducing the spacing size resulted in significantly decreasing ion selectivity and permeation rate. The GO membrane exhibited a lower permeation rate for Ca^2+^ and Mg^2+^ ions than Li^+^, Na^+^, and K^+^ ions, but no identifiable monovalent ion concentration exists. Based on these results, detectable ion transportation can happen when at least three layers of water molecules surround the ions. There is no permeation rate with a d-spacing below 0.7 nm for the GO membranes. In a further work^42^, with no charge density of polyethylene terephthalate (PET) membranes, a substantial increase in Li-ion transport and a considerable loss in Li-ion selectivity was observed when the thickness of the membrane was decreased, and the nanochannels size of PET membrane was increased from 0.6 nm to 1 nm in diameter. Other critical descriptors such as different classes of porous materials, such as the covalent/metal−organic frameworks (C/MOFs) with a narrow distribution of pore sizes are suitable for the ion-selective separation. Angstrom-sized windows and nanometer-sized cavities are the essential parameters for finding a suitable MOF, showing excellent ion selectivity^45–47^. As a result, such geometrical descriptors (size of nanochannels, thickness, distance, and different materials) highly indicate the resulting ions’ selectivity and permeation rate. Hence, it is sensible to utilize these geometrical descriptors for ML.

##### Effect of nanochannel surface charge

In order to achieve practical application, suitable descriptors must be chosen depending on the different situations. Another significant descriptor should be considered when the nanochannel surfaces possess the negative charges introduced through the functional groups on the inner surfaces of the nanochannels. As a result, ion affinity to the functional groups is substantially effective in controlling ion selectivity^48^. Based on the literature, reducing the negative charge density of the functional groups in the nanochannels causes a significant drop in the selectivity of ions and permeation rate^48,49^. According to Wen et al.^49^ it is not clear how the distribution of carboxylate acid as functional groups and their arrangement in the membrane structure can change Li-ion selectivity and transportation rate. In another investigation, Zhao et al.^50^ introduced sulfonate groups between graphene sheets of the rGO-SDDS-rGO membrane that exhibited Li^+^ to multivalent cation selectivity of around five, and poor selectivity of about one for Li^+^ to monovalent cations.

##### Effect of nanochannel morphology

The functional groups onto the nanoscale membranes are not always applicable since other aspects of descriptors, such as morphology and the intrinsic nature of the nanopores, have a significant impact. These are the important contributing factors that need to be considered^51^. To investigate the transport of ions in nano/subnanometer size membrane, morphological defects are important for understanding ion selectivity. Morphological effects such as breaking the symmetry of nanochannels can affect ion conductivity and transportation in nanochannels with the charged surfaces^52,53^.

##### Effect of the driving force

The driving forces, including applied potential, pressure, temperature, and concentration difference, are the primary descriptors for studying ion mobility within nano-scaled membranes. Upon applying the electrical potential between two sides of the nanochannels, increasing the potential and power density causes the enrichment of some ions on one side and depletion of the others on the other side^54^. Therefore, in designing ISMs, the current operating limitation is considered one of the restrictions. Recently, Razmjou et al.^47^ demonstrated that electric field power has a straightforward relationship with the diffusion coefficient in the case of vermiculite membrane with 0.4 and 0.8 nm interlayer spacing. As shown in Table 1, in some studies, the effect of the driving force is considered as an important descriptor of ISMs. Table 1 also presents the descriptors and labels of other important studies on ISMs.

**Table 1:** Different descriptors and labels for the AI methods in ISMs application.

Membranes	Important descriptors	Optimized labels	Ref
PET membranes	∙ Nanochannel size ∙ Nanochannel thickness ∙ Driving forces ∙ pH ∙ Surface charge density	✓ Ion transport rate ✓ Ion selectivity	^43^
ZIF-8 and UiO-66 membranes	∙ Pore size ∙ pH ∙ Applied voltage ∙ MOF chemistry ∙ Ion dehydration mechanism	✓ Ion transport rate ✓ Ion selectivity	^46^
Graphene-based membranes	∙ Interlayer spacing ∙ Sieve size ∙ Membrane’s thickness ∙ Channel size ∙ Temperature	✓ Ion transport rate ✓ Ion selectivity	^46^
2D-Vermiculite membranes	∙ Angstrom-sized windows ∙ Nanometer-sized cavities ∙ Interlayer spacing ∙ Driving Forces ∙ pH	✓ Ion transport rate ✓ Ion selectivity	^47^
PET membranes	∙ Membrane surface charge ∙ Ion type ∙ pH ∙ Subnanometer Pore Size	✓ Ion transport rate ✓ Ion selectivity	^49^
rGO-SDDS-rGO membranes	∙ Channel dimension ∙ Functional groups ∙ Hydrated ion diameter ∙ Ion dehydration energy	✓ Ion selectivity	^50^
Polyimide membranes	∙ Surface charge ∙ Wettability ∙ Pore size	✓ Ion conduction	^53^
MXene	∙ Channel dimension ∙ Charge density ∙ Membrane thickness ∙ Hydration radius	✓ Ion transport rate ✓ Ion selectivity	^112^

#### Energy-based descriptors

Computational simulation methods can evaluate various materials’ properties by performing simulations instead of actual material synthesis^20^. Several factors play significant roles in the design of ISMs through the experimental investigations. Although ion transportation mechanisms inside the nanochannels have been well-studied, other widely descriptors called energy-based descriptors implement the interaction behavior between the membranes and the ions. To provide more information in describing the actual behavior and interaction between the materials and ions for a given framework, energy-based descriptors can enhance the models’ performance using molecular dynamics (MD) simulations and density functional theory (DFT) calculations. Simulations can produce some critical characteristics of the materials without performing the actual experiments. The recent MD simulations exhibited no ion transportation in graphene oxide (GO) membranes^55^ with d-spacing of below 0.7 nm, wherein it was shown that implementing the charged functional groups in GO nanochannels has a negligible effect on the ion transportation and ion selectivity. At the same time, at least two layers of water are essential for ion transportation^55^. DFT modeling was applied to obtain and characterize organic molecules’ quantum mechanical characteristics and electronic properties such as electron affinity, the highest and lowest occupied molecular orbital, reaction properties, structural information, and atom numbers^56^. For instance, Zhang et al.^57^ revealed the mechanism of lithium ions adsorption through λ-MnO2/graphene composite by implementing DFT calculations combined with electrochemical adsorption experiments. DFT was used to calculate the geometric and electronic structure of the composites. Furthermore, DFT calculations were implemented to study electronic conductivity, ionic conductivity, and ion selectivity. Sendek et al.^58^ investigated the solid lithium conducting materials with fast single-crystal Li-ion conductivity using the DFT simulations guided by the ML-based methods. Good prediction results and in-depth understanding were possible. Table 2 outlines studies with energy-based descriptors used for AI methods in ISMs.

**Table 2:** Different descriptors for energy-based AI models.

Application	Membranes	Important descriptors	Labels	Ref.
MD	PET membranes	∙ Average pore radius ∙ Electrostatic interaction	✓ Ion transport rate ✓ Ion selectivity	^42^
MD	-	∙ Pore size ∙ Energy barriers ∙ Ion type ∙ Membrane surface charge ∙ Ion dehydration mechanism	✓ Ion transport rate ✓ Ion selectivity	^43^
MD	2D-Vermiculite membranes	∙ Channel dimension ∙ Inter-surface distance ∙ Driving forces	✓ Ion transport rate ✓ Ion selectivity	^47^
MD	Graphene oxide (GO) membranes	∙ Channels dimension ∙ Different layers of water ∙ Ionic charge	✓ Ion transport rate ✓ Ion selectivity	^112^
DFT	Manganese oxide (λ-MnO2)/graphene	∙ Electronic conductivity ∙ Band gap ∙ Degree of hybridization ∙ Diffusion energy barrier	✓ Ion selectivity ✓ Ion conductivity	^56^

#### AI tools for modeling the ISMs for ions recovery

**Fig. 3:**
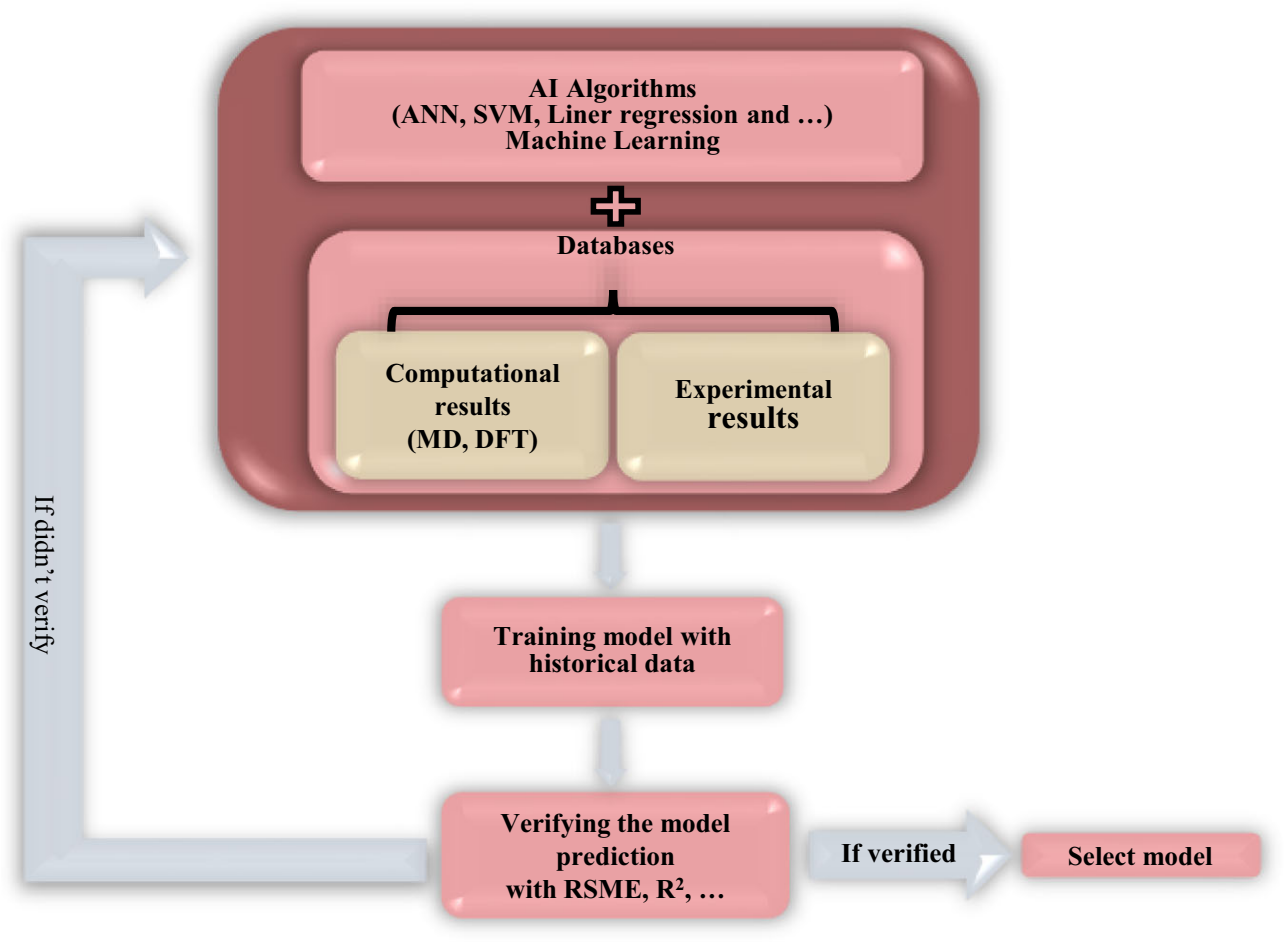
The roadmap of selection AI algorithms for ISMs discovery. The validation criteria in this roadmap are parameters such as R-squared and Root-mean-square deviation (RMSE), which show the accuracy of the models. AI algorithms should be able to create models that have good accuracy based on experimental and computational datasets. Part of the dataset is used to train the model and part of it is used to test and validate the model. Validated models that have good accuracy can be used for ISMs discovery.

Apart from the traditional one-by-one theoretical, experimental, and computational material simulation methods, AI could be a great approach that can accommodate the massive data requirement for material design, discovery, and its challenges. Screening the large material design, processing the material’s characterization, reducing the prediction time of simulations, analyzing the characterization dataset, predicting the property of complex material systems, mapping accurately to the multi-dimensional synthesis recipes of materials, handling huge amounts of data, and extracting the significant scientific principles and intrinsic information from different material designs, are the reasons why AI is accurately applicable in material design^20,59^. All of the information mentioned above can contribute to the dataset of the high volume of experimental and computational findings. By applying the suitable ML methods, all of the valuable information of one data point can be analyzed and discovered in a significantly lesser time^20^. However, the AI has made no effort to predict ISMs, including selectivity and membrane performance. The accuracy of AI is highly dependent on the quality and quantity of data, requiring high volume data mining due to lack of standardized databases on membranes. Given the high number of parameters and the complexity of the membrane processes, ML approaches can be valuable, which are increasingly applied in many complex systems. Therefore, implementing MI and AI to establish a specified platform for membrane design is necessary. Figure 3 shows a roadmap for selecting the appropriate AI algorithm to have an accurate model.

Bowen et al.^60^ developed a single artificial neural network to predict the performance of nanopore membranes by investigating the effect of charge, steric hindrance, dielectric, transport effect, ions concentration, composition, pH, and operating pressure as the primary descriptors, and the results agree with the experimental data. In another investigation, Darwish et al.^61^ implemented the ANN model to predict the rejection of Na^+^ and Mg^2+^ ions through the nanopore membranes at different concentrations and pressures to demonstrate that the ANN model can predict experimental results successfully and the ANN could successfully predict the nonlinear behavior of rejection vs. pressure and flux. Fu-HengZhai et al.^62^ developed a protocol for designing anion exchange membranes (AEMs) with predictable OH^-^ conductivity using deep learning. They were able to predict the conductivity of OH-ions with poly (2,6, dimethyl phenylene oxide)-based AEMs grafted with a cationic group. In another investigation, the ML-based prediction model was used with the DFT-MD simulations to discover many new solid materials to predict superionic Li-ion conduction^59^. DFT was used to obtain the electrochemical properties of molecular materials with very accurate results. However, DFT computations may not be precise for high-throughput screening since they may take longer. Allam and Cho et al.^56^ tried to facilitate the design of carbon-based molecular materials through the DFT-ML framework by developing a high-throughput screening method.

## ML capabilities for the analysis of ISMs

The ML attempts to understand the hidden laws and relationships between the groups based on the previous information and classify them into individual groups^63^. Experimental data and simulation results may be obtained from the dataset. For the learning process, various ML methods enclose multiple algorithms such as supervised (including regression and classification), unsupervised (e.g., K-nearest neighbors and principal components analysis), and reinforcement learning algorithms (e.g., Q-learning and Markov decision process)^64^. In the following, we discuss these individually.

### Modeling of the ISM

Intelligent methods and especially the ML techniques are able to model, select effective parameters and even optimize them to boost the performance based on a wide variety of datasets from the various regions of membrane technology. The ML and its subclasses can step into the post-treatment process and even the design of selective membranes. It is worth mentioning that similar studies have been carried out to model the proton exchange membranes that can be instructive for future studies for ISM investigations^65–67^. ISMs are the main parts of ion-selective electrodes (ISEs), mostly polymeric plasticized membranes^68^. In this regard, the search for ligands with higher selectivities towards the ions is essential. Recently, quantitative structure-property relationship (QSPR) modeling has been utilized to accelerate the discovery of new ligands for Mg^2+^/Ca^2+^^69^ and Li^+^/Na^+^^70^ selectivities. The applied methods have shown the ML capabilities in discovering and predicting the new materials with better performance. In another study^71^, a supervised ML algorithm based on multilayer perceptrons (Adam optimization^72^) was trained using a combinatorial database including experimental and computational fluid dynamics (CFD) simulation results, where the authors claimed that the method could accelerate the parameter fitting process as valuable information for understanding the kinetics and thermodynamic parameters of ISMs. Using machine learning and deep learning, one can create a new framework for a deeper understanding of membrane–solvent interactions by visualizing the effect of different solute functional groups on rejection. This enables the design of membranes with improved selectivity^73,74^.

### Optimization

**Fig. 4:**
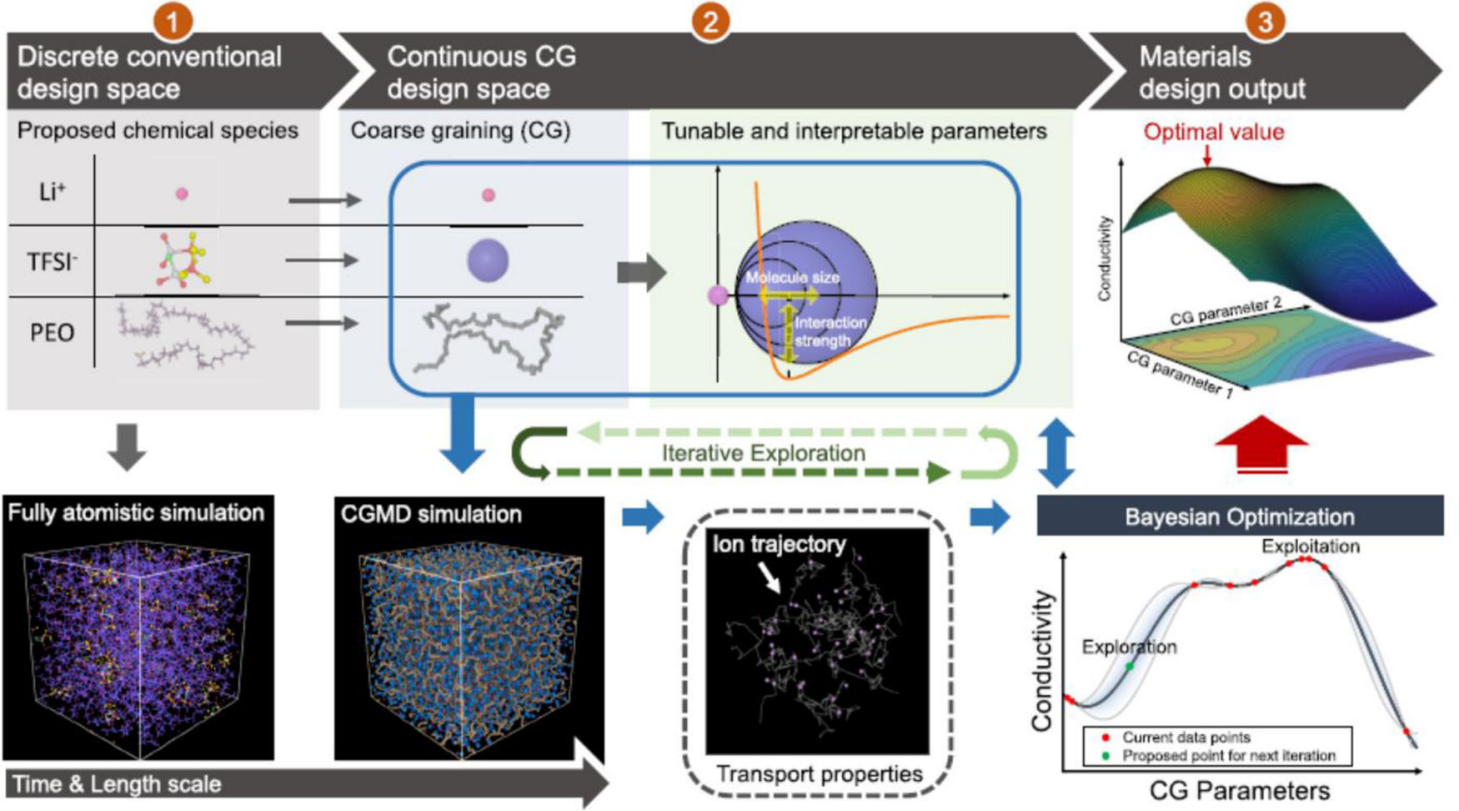
Schematic illustration of the coarse-grained MD-Bayesian ML method for optimizing conductivity of polymer electrolytes (reproduced from Wang et al.^75^). In this approach, using the coarse-graining process, new materials are designed (stage 2) using the discrete chemical species (anions, backbone polymers, and secondary site of polymers) obtained from stage 1. Afterward, the relationship for properties was predicted using the Bayesian optimization (BO) in stage 3. Reprinted with permission from^75^. Copyright 2020 American Chemical Society.

The ultimate goal of every membrane process is to optimize the determinant variables in the trade-off between selectivity and permeability to reach a high separation ratio. The effective parameters can be detected in the membrane materials to process conditions. In an interesting report, Wang et al.^75^ using the results from coarse-grained MD simulation coupled with Bayesian optimization (BO) method (Fig. 4) determined the optimal conductivity of solid polymer electrolytes for Li ion. Figure 4 shows the results of this work. Using the expandable (to micro properties) CGMD-BO model, they reduced the need time to minutes for obtaining the desired conductivities that are dependent on all the molecular properties of the anions (anion size, salt concentration, and anion involved vdW interaction strengths), backbone polymer chain (monomer size and polymer involved nonbonding interaction strengths) and secondary sites of the polymer chain (molecule size and secondary site involved nonbonding interaction strengths). The authors claimed that the trained model could accurately predict the conductivities of the common electrolytes.

### Reduction of computational cost using ML

One of the important features of ML techniques in ISMs is ML-driven molecular and atomic simulations to discover the new ISMs. ML can assist the molecular dynamics simulations, while the DFT provides initial screening and predictors to reduce the simulation consumption. For example, Sendek et al.^58^ screened 21 candidates from a large material dataset (12,000+) for solid-state lithium conductors. They developed a classification model using logistic regression based on the published data in the literature. Two main conductors were detected as nitride- and oxygen-based materials. The comparison between the randomly chosen and ML-selected materials represents that ML-selected samples could also be conductors.

### Prediction

As mentioned previously, ML methods can predict various aspects of membrane technology, such as membrane fouling^76,77^ and the lifetime of a membrane^32^. Liu et al.^33^ collected 1815 vectors for PVDF/PES/PSF membranes and considered various features such as basic materials (including polymer and solvent), membrane fabrication (containing phase inversion technique, exposed time, relative humidity, the thickness of the wet membrane, casting and coagulation temperatures, and non-solvent), membrane structure (including thickness, porosity, surface contact angle, and roughness), and operation (operating pressure and separating substances properties such as partial charge, molecular weight, radius, and concentration) to predict the membranes performances with Random Forest (RF) algorithm. The results showed that the fabrication of membranes for salts is more complicated than for the macromolecules. The same authors previously conducted another study for the combination of 166 hydrophilic and 175 hydrophobic monomers to predict hydrocarbon-based sulfonated copolymers as proton exchange membranes and designed four novel copolymers to predict their performance better than the Nafion 117. Similarly, AI methods have been used for ion transport in polymeric electrolytes, which can be enlightening for investigations on ion transport through ISMs. In a series of studies^78–80^, important and effective parameters were identified in the final properties of the Li selective electrolyte polymer blend. The objective function was defined considering the molecular weight of the polymers and their compositions, polarity, and compatibility as the variables. The Bayesian ML method, in combination with MD simulations, was used to predict the trade-off between ion transport and mechanical properties. Additionally, the authors trained the CNN model data obtained from the coupled Kinetic Monte Carlo (KMC) simulations for the nanoparticles’ diffusivity^81^. The results showed that a data-driven approach could predict ion transport for a wide range of nanoparticle microstructures.

### Cross-validation

To prevent overfitting in the process of ML algorithms, it is necessary to use cross-validation methods, which prevent the incorrect predictions by an ML algorithm. Therefore, if cross-validation is not used to improve the training quality of ML models, these models cannot make accurate predictions, and the results obtained from these methods are not reliable. One of the most widely used methods is k-fold cross-validation, in which input data are divided into several parts, and the desired model is taught using an amount of data. Finally, the accuracy of the model in predicting the selected parameter is checked using the other part. In addition to ensuring the predictions made, this is also used to optimize the model parameters. For example, cross-validation leads to optimizing the number of neighbor points required in the k-nearest neighbor classifier algorithm. Therefore, the predictions made by this algorithm have the slightest deviation from the actual data^82–84^.

## Limitations of AI in the design of ISMs

AI has the ability of a computer to think and learn and is a major breakthrough in technology due to the increase in the computing power of computers. The increasing use of AI technology in various fields is undeniable^13,85–87^. With the help of AI algorithms, different data patterns can be examined and used to modify the structure and function of different materials. In fact, with the help of AI, the properties of various materials, including ISMs, can be engineered. However, AI algorithms examine a large number of data and discover specific trends and patterns unknown to humans. Despite all the advantages and popularity of these methods, the use of these algorithms is limited. If not taken into account, there are important points that will cause a lot of errors in the calculations, and in this regard, AI algorithms need a large and high-quality dataset^88–90^.

### Lack of big data for modeling of ISMs

The quality and performance of AI algorithms depend on input data because AI uses the existing information to learn, and they require large educational datasets. In AI algorithms, data is used to train and test the algorithms. If the amount of data used is small, AI algorithms can cause significant errors in predictions due to a lack of proper learning of AI algorithms in the absence of sufficient data. Suppose the amount of data used to predict the various properties of materials is small; in that case, AI algorithms cannot draw comprehensive patterns, which leads to unrealistic predictions of the properties of new compounds and thus may not achieve the desired goal. Using different datasets that have made the properties of other materials available to the public can largely solve the lack of data in developing the use of AI to design and build ISMs.

### Lack of atomistic properties data

The problem of AI algorithms that causes unrealistic and erroneous predictions is not considering different materials’ atomistic and quantum properties for designing and constructing ISMs. Since different molecular and quantum properties such as bandgap, electronegativity, atomic radius, valance electrons, etc., determine many properties of the materials, their non-use in AI algorithms creates unrealistic relationships between the data. As a result, errors occur in the predictions. These algorithms can examine the data to design and develop comprehensive and deep relationships between various properties of the materials and their properties. As a result, the errors resulting from the predictions made by AI algorithms are minimized and the projections made are closer to reality. The use of atomistic and quantum data provides the best conditions for the use of AI in the designing and manufacturing of ISMs, which will be discussed in the next section.

## The role of computational chemistry in AI-based engineering of ISMs

**Fig. 5:**
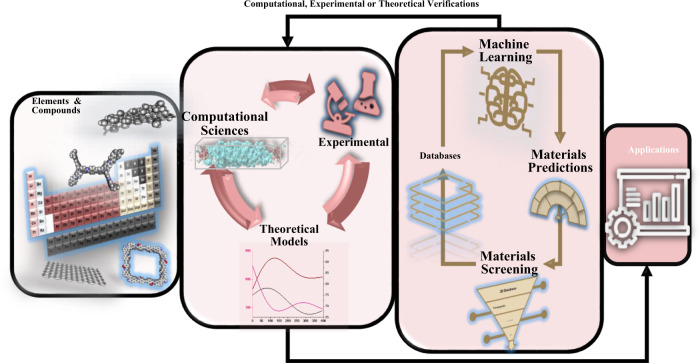
Using computational and experimental data for Ai-based material discovery. Computational chemistry can improve the datasets used in material discovery with theoretical studies, and along with the properties that can be observed in the laboratory, atomic properties (which can be studied in computational chemistry) can also be entered into the dataset. In this way, the accuracy and reliability of the AI-based material discovery will increase^92^.

Computational chemistry is important in investigating materials and designing ISMs^51^ since the computational chemistry methods provide properties of materials that are impossible to achieve via experiments. Using these methods, one can access more features of the materials. Given that the use of AI and ML requires a large amount of data, using data existing in this field is an important part of the AI to data analysis^91^. Different databases such as AFLOWLIB, Pymatgen and Materials Project are needed to teach AI models to predict the properties of membranes correctly, and consequently select the optimal membrane to separate the ions. These databases can include data related to the band gap, electronegativity, atomic radius, valance electrons, etc. In addition to databases, open access codes such as NanoHUB^36^ that have been used before and have shown good results can be used to achieve more accurate outcomes and prediction. Figure 5 shows how computational and experimental methods or theoretical verifications can relate to methods based on AI. Many compounds can be prepared by combining atomic elements, which can be studied experimentally, theoretically, or computationally. The results of these experiments and computational or theoretical methods can provide us with a vast set of data. By screening this data and using the ML, materials with unique properties can be identified and selected^92^.

### Improving membrane designing using advanced computational methods (MD, DFT, etc.)

The MD and DFT calculations provide a series of molecular information related to atomic properties^93^. This type of information is not available through experimental tests or AI. Therefore, this method makes it possible to have a deeper and more accurate ISMs design by considering the quantum and atomic properties^94^. Given that AI has limitations, such as the inability to observe quantum properties, the use of MD and DFT can overcome these limitations to an acceptable level. AEM is one type of ISMs^95^. Computer simulation is one of the main tools for studying the microscopic interaction of cationic groups in AEM systems^96^. Recently, Chen et al.^97^ investigated the effect of different cations and their structural properties on water absorption in AEM using MD and DFT. This study showed that the structure of cationic groups has a significant effect on water absorption of the AEM. Also, it was shown that creating a balance between ion transfer and dimensional stability is alone not possible by merging the cationic groups.

### Assisting computational methods by ML

In this section, the application of ML to improve the conventional DFT methods is investigated. For this purpose, we should develop theoretical points and secondly the practical application as well as the positive effect of applying this novel idea^98^.

#### Improvement of DFT performance by utilizing ML from a theoretical viewpoint

DFT is one of the typical computational methods based on quantum mechanics used in multi-electron systems studies^99^. One of the most important strengths of DFT is high accuracy in relevant calculations, which are widely used in chemical analyses. Undoubtedly, the complexity of the computational methods and the occupation of large volumes of resources by the processes of these complex calculations are the main limitations of DFT calculations. DFT is based on non-classical electron interaction, which can be a limitation of this method^100^.and using DFT for various applications, including material discovery and ISMs design, requires relatively high cost and time. Therefore, researchers seek to replace part of the computational process performed by DFT with AI such as ML. Thus, using ML can be an excellent approach to improve some DFT defects. DFT calculations can be reproduced using the ML if sufficient data are available. By doing this, the deviation of DFT values from the deviation of the results of the DFT calculations from the experimental values will be more compact^92,101,102^. However, an acceptable amount of data is required to use ML, which is one of the important limitations of using ML. Nowadays, there are many ambiguities in this area, and much research is needed^103^. The study of this issue from a theoretical point of view was done by Ramprasad^104^, and the compatibility of ML-based data prediction with the leading scientific processes was confirmed^104^. To conclude, the observations and data are analyzed at the beginning of the approach. Then a prediction can be made according to the previous behavior. Finally, a quantitative theory is presented according to the observations made. Therefore, it makes sense to use ML to advance DFT calculations^104^.

#### Improvement of DFT performance by utilizing ML

Molecular simulations in large-scale and long-time systems similar to experimental conditions are not possible or costly. Recently*,* Pattnaik et al.^105^ used deep learning (DL) to simulate large systems using the data from DFT on small systems. The forces predicted in molecular simulation assisted with ML can be calculated accurately by the qualitative dynamic properties of materials. Diffusion describes random particle motion and has an important influence on determining the functionality of materials^106^. Recently, diffusion mechanisms were examined by Elbaz et al.^106^ using the DFT and MD simulations. In continuation of this research, an attempt was made to overcome the limitations of the usual computational methods by AI and ML to conclude that the use of AI could provide a platform for the investigation of diffusion mechanisms to be predicted fully automatically using the existing datasets. Thus, combining ML with DFT can reduce the problems encountered in the conventional DFT methods, including computational complexity and related costs. However, the accuracy of this novel method is much lower than expected^107^, and the combination of ML with DFT can cause fundamental changes in material science research (such as material discovery and fabrication of ISMs)^108^.

### New ISMs development using AI

Nowadays, AI is widely used in materials science and sub-disciplines such as material discovery and membrane fabrication^109^. Due to computational and laboratory methods development, a considerable amount of data with different complexity, quantity, and quality is being produced today. It is vital to maintain and interpret such a considerable amount of data and advance materials science. Still, researchers have been using various ML algorithms to define the patterns and relationships from such vast data^92^. Some sub-branches of AI and their use in materials science and membrane fabrication are introduced in the following sections.

#### Inverse design to achieve custom components

The inverse design aims to find a substance or product with specific characteristics. Inverse design is generally different from forwarding development. In the conventional method, the target material is produced during experiments, and its properties are further investigated. However, the inverse design starts from the end. The desired properties of the material are given as input, and the corresponding material with these properties is suggested^110^. This process can be difficult, and an optimal solution may not exist, but one or more solutions may be offered^110^.

#### Use computer vision to analyze membrane images

Computer vision in an AI system extracts data and information from images. But today, it is used as an interdisciplinary technology in various fields of science^111^.

#### Screening and big data

**Fig. 6:**
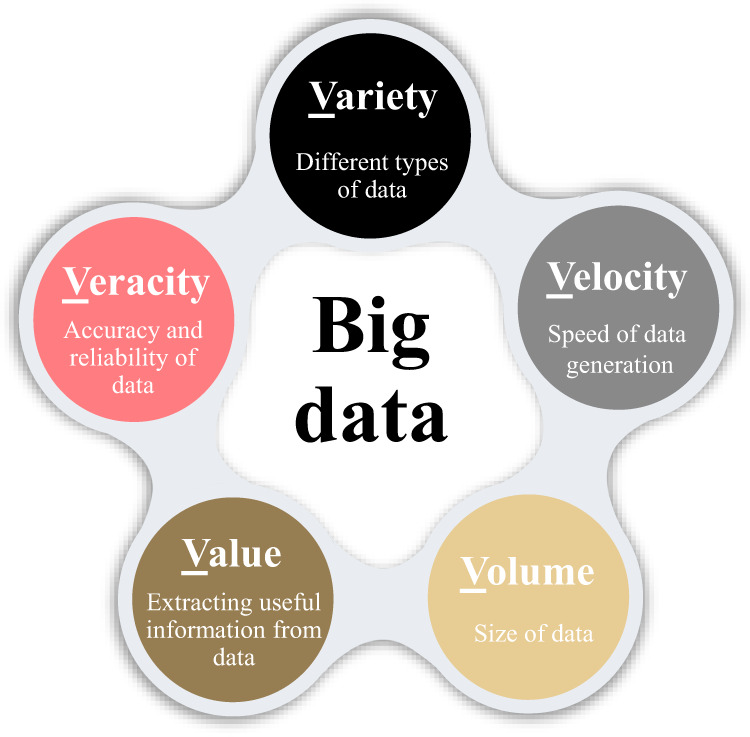
Five main features of big data. These features, are known as the ‘Five V’s’. Big data is described by ‘Five V’s’ including: Value, Volume (size of data), variety (diversity of data), and veracity (Data accuracy and reliability (, and velocity (speed of the data gathering)^92^.

High-throughput screening uses a huge amount of data to perform computational work and to identify the properties of materials and the design of the target material. Big data is a research method that extracts high volume and complexity data. Due to its high complexity, this volume of data is usually not easily analyzed and parsed. The five main features of big data are volume, velocity, variety, veracity, and value (Fig. 6), known as the *‘five V’s’*^92^. Therefore, in ISMs, the target membrane can be fabricated using these two ideas. Utilizing two sewing processes is very important for using ML to design and select materials. ML is one of the branches of AI that can automatically learn a pattern using the data in a field and use this pattern in different situations to provide the appropriate answer based on the new dataset^97^. Table 3 provides a brief description of the recent articles.

**Table 3:** A brief explanation of the previous works for the application of AI methods in the discovery of Li-ISMs.

Heading	Type and model of computational chemistry calculations and AI	summery	The most important result	Ref
Improving membranes design using advanced computational methods (MD, DFT, etc.)	MD and DFT	∙ water absorption on AEM	✓ structure of cationic groups has a significant effect on water absorption on AEM	^97^
	DFT	∙ modification of a layer of ion-imprinted polymer to the PVDF (Poly vinylidene fluoride) membrane with a molecular-scale design	✓ molecular-level design with DFT can increase ion-ion selectivity in membrane construction	^113^
	DFT	∙ diffusion mechanism of hydroxide ions and protons along the water wires	✓ electronic structure has an important effect on the water wire conductivity in the classical nuclei simulations	^114^
Improvement of DFT performance by utilizing ML	DL and ML	∙ simulate large systems using data from DFT on small systems	✓ Forces predicted by ML in molecular simulation can be calculated accurately by qualitative dynamic properties of materials	^105^
	MD and AI	∙ Examine diffusion mechanisms using common computational methods such as DFT calculation and MD and Eliminate their limitations by AI and ML.	✓ AI can predict diffusion mechanisms completely automatically using existing datasets in these fields	^106^

## Outlook

Membranes are selective barriers that separate compounds with different physical or chemical properties. Usually, mass transfer and separations are usually based on membrane due to many advantages such as no phase change, no additives, low energy consumption, and the compact equipment membrane that can occupy a small space. Membranes and membrane processes play an important role in the sustainable development of numerous fields such as energy, environmental management, human health, etc. Membranes are typically determined by their permeability and selectivity. Permeability and selectivity depend on the size of the pores and the properties of the membrane surface. Increasing the pore size enables the membrane to increase permeability and determines the membrane selectivity based on particle size. At the same time, membrane’s selectivity depends on both the surface charge and the membrane composition. To improve the efficiency of membranes, especially in designing and discovering new ISMs with higher selectivity and permeability towards the desired ions, membranes’ surface and molecular structure can be engineered. With the help of AI and the use of data obtained from previous studies and published data about the unique properties of different materials, the membrane performance can be further improved.

ML algorithms use statistics to find patterns within a large volume of data, including numbers, words, pictures, etc. Using ML to search for new materials is energy and time efficient, with an added advantage of processing and analyzing data that could not be performed by normal systems. This method finds the relationships between the structure of the material and its functional properties. Therefore, AI and ML reduce the number of experiments and simulations to achieve the most efficient membrane for ion extractions. Since data is the most important part of any ML model, the quantity and quality of this data can greatly impact the accuracy of predictions and the output of ML. By using highly sophisticated data such as molecular and quantum properties obtained from computational chemistry, the accuracy of the predictions can be increased, and the results can be assured. Previous studies have shown that the use of AI in engineering and improving the efficiency of membranes has been very effective. This expands the use of AI in the design and fabrication of membranes.

## Data Availability

No datasets were generated or analyzed during the current study.
